# Facilitation of Gastrointestinal (GI) Tract Microbiome-Derived Lipopolysaccharide (LPS) Entry Into Human Neurons by Amyloid Beta-42 (Aβ42) Peptide

**DOI:** 10.3389/fncel.2019.00545

**Published:** 2019-12-06

**Authors:** Walter J. Lukiw, Wenhong Li, Taylor Bond, Yuhai Zhao

**Affiliations:** ^1^LSU Neuroscience Center, Louisiana State University Health Sciences Center, New Orleans, LA, United States; ^2^Department of Ophthalmology, Louisiana State University Health Sciences Center, New Orleans, LA, United States; ^3^Department of Neurology, Louisiana State University Health Sciences Center, New Orleans, LA, United States; ^4^Department of Pharmacology, School of Pharmacy, Jiangxi University of Traditional Chinese Medicine (TCM), Nanchang, China; ^5^Department of Anatomy and Cell Biology, Louisiana State University Health Sciences Center, New Orleans, LA, United States

**Keywords:** Alzheimer’s disease (AD), brain microbiome, dysbiosis, gastrointestinal (GI) tract, lipopolysaccharide (LPS), neurofilament light (NF-L), synapsin-1 (SYN1), the thanato-microbiome (the post-mortem microbiome)

## Abstract

Human gastrointestinal (GI)-tract microbiome-derived lipopolysaccharide (LPS): (i) has been recently shown to target, accumulate within, and eventually encapsulate neuronal nuclei of the human central nervous system (CNS) in Alzheimer’s disease (AD) brain; and (ii) this action appears to impede and restrict the outward flow of genetic information from neuronal nuclei. It has previously been shown that in LPS-encased neuronal nuclei in AD brain there is a specific disruption in the output and expression of two AD-relevant, neuron-specific markers encoding the cytoskeletal neurofilament light (NF-L) chain protein and the synaptic phosphoprotein synapsin-1 (SYN1) involved in the regulation of neurotransmitter release. The biophysical mechanisms involved in the facilitation of the targeting of LPS to neuronal cells and nuclei and eventual nuclear envelopment and functional disruption are not entirely clear. In this “Perspectives article” we discuss current advances, and consider future directions in this research area, and provide novel evidence in human neuronal-glial (HNG) cells in primary culture that the co-incubation of LPS with amyloid-beta 42 (Aβ42) peptide facilitates the association of LPS with neuronal cells. These findings: (i) support a novel pathogenic role for Aβ42 peptides in neurons *via* the formation of pores across the nuclear membrane and/or a significant biophysical disruption of the neuronal nuclear envelope; and (ii) advance the concept that the Aβ42 peptide-facilitated entry of LPS into brain neurons, accession of neuronal nuclei, and down-regulation of neuron-specific components such as NF-L and SYN1 may contribute significantly to neuropathological deficits as are characteristically observed in AD-affected brain.

## Overview

The highest known density of microorganisms anywhere in the biosphere is in the human GI-tract microbiome at about ~10^11^ microorganisms per gram of GI-tract content (Angelucci et al., 2019; Castillo-Álvarez and Marzo-Sola, 2019; Fox et al., 2019). This vast number represents a remarkably complex and highly dynamic source of microbes; of the approximate ~1,800 different microbial phyla that make up the GI-tract microbiome, the overwhelming majority are facultative anaerobic bacteria with archaea, fungi, microbial eukaryotes, protozoa, viruses, and other microbes making up the remainder (Bhattacharjee and Lukiw, 2013; Fox et al., 2019; Tierney et al., 2019). One major species of bacteria in the human GI-tract microbiome, about ~100-fold more abundant than *Escherichia coli* in certain GI-tract regions is *Bacteroides fragilis*, an obligate, anaerobic, non-spore forming Gram-negative, rod-shaped enterotoxigenic bacterium. *B. fragilis*: (i) generates a remarkable array of highly neurotoxic exudates; and (ii) produces a particularly virulent, pro-inflammatory LPS glycolipid subtype (BF-LPS) that accumulates in Alzheimer’s disease (AD) brain (Sears, 2009; Fathi and Wu, 2016; Lukiw, 2016a,b; Wexler and Goodman, 2017; Zhao et al., 2017a,b,c; Allen et al., 2019). Besides BF-LPS, *B. fragilis*-derived neurotoxins include small non-coding RNA (sncRNA), bacterial amyloids, endo-, exo-, and enterotoxins such as *fragilysin*, and truncated LPS molecules known as lipooligosaccharides (LOS). These neurotoxins have recently been shown to be capable of transversing normally restrictive gastrointestinal (GI) tract and blood-brain barriers (BBBs) in transgenic murine models of AD (Varatharaj and Galea, 2017; Sweeney et al., 2018; Tulkens et al., 2018; Barton et al., 2019; Erdö and Krajcsi, 2019; Panza et al., 2019; Sweeney and Lowary, 2019). Both the GI-tract and BBB may become weakened with aging or following surgery, disease or trauma (Sweeney et al., 2018; Sweeney and Lowary, 2019). For example, BF-LPS and the *B. fragilis*-derived enterotoxin *fragilysin* very effectively disrupt cell-cell adhesion, in part by E-cadherin cleavage and/or the action of LPS binding protein and Toll-like receptor 4 (TLR4), and subsequent LPS internalization, followed by translocation of neurotoxins into the systemic circulation, past the BBB and on into the parenchyma of the brain [Wu et al., 1998; Holton, 2008; Tsukamoto et al., 2018; Barton et al., 2019; Jeon et al., 2019; Lukiw, 2019 (submitted)].

## LPS Accumulation in AD Brain

**Figure 1:**
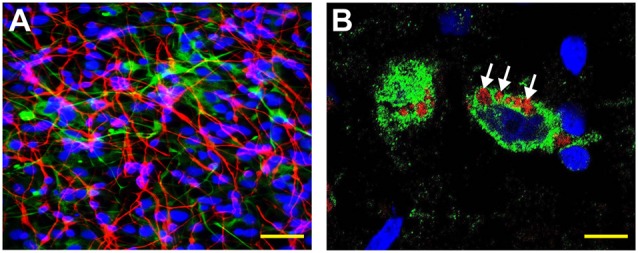
Human neuronal-glial (HNG) cells (transplantation grade) in primary co-culture were used to study the dynamics of amyloid-beta 42 (Aβ42) peptide-mediated entry of lipopolysaccharide (LPS) into neurons (Bhattacharjee and Lukiw, 2013; Zhan et al., 2018; Zhao and Lukiw, 2018a,b; Zhao et al., 2019). **(A)** HNG cells are a primary co-culture of neuronal [β-tubulin III (βTUBIII)-stained; red; *λ*_max_ = 690 nm] and glial (GFAP-stained; green; *λ*_max_ = 520 nm) human brain cells; HNG cells are also stained for nuclei (DAPI-stained; blue; *λ*_max_ = 470 nm); cells shown are ~2 weeks in culture; HNG cells are about ~60% neurons (red) and about ~40% astroglial (green) at ~65% confluence; human primary neuronal and glial “support” cell co-cultures are utilized, because human neuronal cells do not culture well by themselves (Cui et al., 2010; Zhao et al., 2017c); HNG cells were exposed to 50 nM LPS for 36 h in the presence or absence of 10 nM Aβ42 peptides; other LPS concentrations at similar times displayed analogous trends; yellow scale bar (lower right) ~50 μm. **(B)** Affinity of LPS for the neuronal nuclear envelope (white arrows); LPS (red; *λ*_max_ = 690 nm); β-tubulin III (βTUBIII)-stained (green; *λ*_max_ = 520 nm) and nuclei (blue; *λ*_max_ = 470 nm) stained HNG cells; white arrows indicate punctate and perinuclear clustering of LPS and LPS affinity for the nuclear envelope as has been previously reported (Hill and Lukiw, 2015; Zhan et al., 2016, 2018; Yang and Chiu, 2017; Zhao et al., 2017a,b); yellow scale bar (lower right) = 20 μm.

Multiple, independent research laboratories have reported: (i) the association of LPS and microbial-derived amyloid with AD brain (Zhao and Lukiw, 2015; Zhao et al., 2015); (ii) the remarkable affinity of specific LPS isoforms with AD brain parenchyma (Lukiw, 2016a,b); (iii) that Gram-negative bacterial molecules associate with AD neuropathology (Zhan et al., 2016); (iv) that microbiome-derived *E.coli* LPS and *B. fragilis* LPS associate the hippocampal CA1 region of AD brain (Zhao et al., 2017a,b,c); (v) of LPS accumulation within neocortical neurons of the AD brain that impair transcriptional output (Zhao et al., 2017a,b,c); (vi) that there is a strong association of LPS with neuronal nuclei and the specific LPS-mediated impairment of expression of the neurofilament light (NF-L) chain gene expression (Lukiw et al., 2018); (vii) of LPS association with the amyloid plaques, neurons and oligodendrocytes in AD brain (Zhan et al., 2018); and (viii) a significantly reduced expression of the AD-relevant synaptic components such as synapsin-1 (SYN1) in LPS-treated human neuronal-glial (HNG) cells in primary culture (Zhao et al., 2019). Most recently, it has been shown that LPS has a very strong affinity for, and association with, the neuronal nuclear envelope of the HNG cells in primary culture. This is also observed in the superior temporal lobe neocortex (Brodmann area A22; Wernicke’s area) and the hippocampal CA1 region of AD-affected brain (Lukiw, 2016a,b; Zhao et al., 2017a,b,c, 2019; Lukiw et al., 2018; Ticinesi et al., 2019; Figure 1). Interestingly in moderate-to-late-stage AD LPS totally encapsulates neuronal nuclei in the AD brain with the subsequent restriction in the output of genetic information from those neuronal nuclei (Lukiw et al., 2018; Zhao and Lukiw, 2018a,b; Zhao et al., 2019). Interestingly, gene expression profiling showed a long-lasting deficit in neuron- and synaptic-specific gene expression and signaling in the hippocampus and neocortex of both transgenic murine models for AD and in patients with mild cognitive impairment or AD (Colangelo et al., 2002; Counts et al., 2014; Jaber et al., 2019; Parra-Damas and Saura, 2019).

## Aβ42 Peptides and LPS in AD Neurons

**Figure 2:**
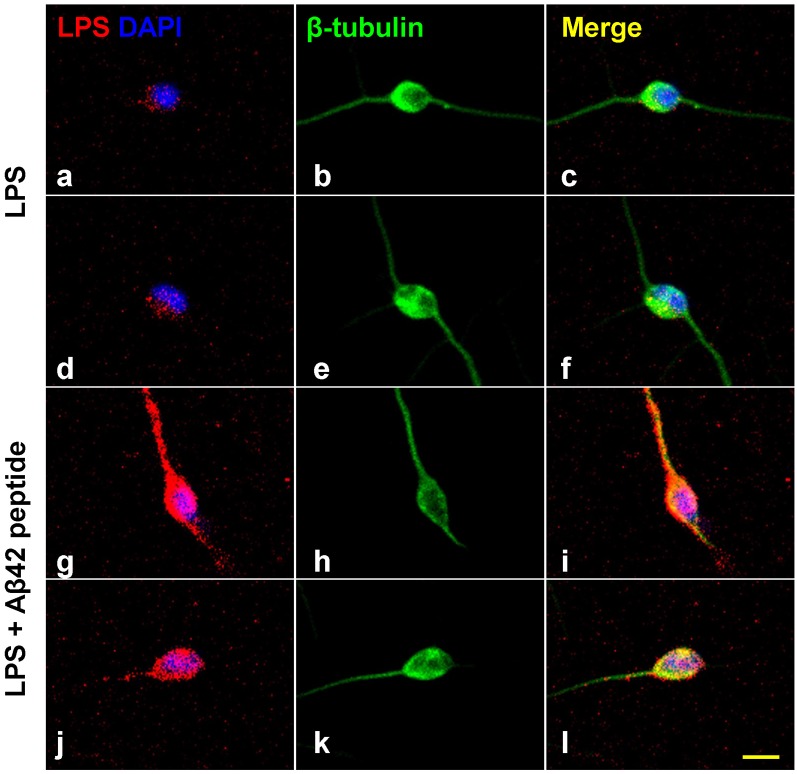
Increased affinity of LPS for neuronal nuclei in the presence of Aβ42 peptide. Panels **(A–L)** show LPS-neuronal interactions in the presence or absence of Aβ42 peptide; **(A–F)** in the absence of Aβ42 peptide and **(G–L)** in the presence of Aβ42 peptide. LPS preferentially associates with human neuronal nuclei both in Alzheimer’s disease (AD) and in LPS-addition experiments (Zhao et al., 2017a,b,c; Zhao and Lukiw, 2018a,b). Panels **(A,D)** show LPS (red) affinity for a polar region of a single DAPI-stained neuronal nucleus (blue). Panels **(B,E)** show single neuronal nucleus stained with neuron-specific β-tubulin III (green). Panels **(C,F)** show merged stain indicating LPS affinity for the polar region involving a single DAPI-stained neuronal nucleus. Panels **(G,J)** show the presence of Aβ42 significantly increases the affinity of LPS for single DAPI-stained neuronal nucleus. Panels **(H,K)** show single neuronal nucleus stained with neuron-specific β-tubulin III (green). Panels **(I,L)** show merged stain indicating LPS affinity for the neurite and soma of a single DAPI-stained neuronal nuclei. The results suggest that LPS is stimulated to associate with DAPI-stained neuronal nuclei in the presence of the hydrophobic Aβ42 peptide; neither Aβ40 peptide or β-actin showed comparable “association” effects (Zhao et al., 2017a,b,c; Lukiw et al., 2018); yellow scale bar (lower right) = 50 μm.

Recent evidence shows that LPS-type glycolipids not only have an unusually high affinity for neuronal nuclear membranes but also that amyloid-β 42 (Aβ42) peptides significantly facilitate LPS access and translocation into human neuronal cells and across neuronal nuclear envelopes in HNG cells in primary co-culture (Figures 2A–L). There are several possible explanations for the facilitation of LPS entry into, and their association with, neuronal membranes and neuronal nuclei by Aβ42 peptides or Aβ42 oligomers and these include:

### Pore Formation by Aβ42 Peptides

Amyloid peptides (both Aβ40 and Aβ42) form a remarkable number of heterotypic structures and configurations under pathological conditions. These include: (i) the self-assembly and deposition of multiple types of fibrillar and globular structures and polymorphic assemblies both in solution and on membrane surfaces; and (ii) the formation of heterogeneous ionic pores spanning the lipid bilayer that is linked to the pathogenicity of these molecules. These later findings are supported by multiple independent reports regarding the capability of Aβ42 peptides [2 hydrophobic amino acid residues (isoleucine and alanine) longer (at the C-terminal) than Aβ40] to form up to 2.4-nm diameter pores through lipid bilayer membranes (Lashuel et al., 2002; Connelly et al., 2012; Sciacca et al., 2012; Ullah et al., 2015; Di Scala et al., 2016; Jang et al., 2016; Davidson, 2019; Hicks et al., 2019; Nguyen et al., 2019; Sun et al., 2019; Österlund et al., 2019). Interestingly, the slightly longer and more hydrophobic Aβ42-based peptide assemblies in oligomeric preparations have been observed to form voltage-independent, non-selective ion channels in contrast to Aβ40 peptide-based oligomers, fibers, and monomers which do not generally support pore structure formation (Bode et al., 2017, 2019; Nguyen et al., 2019). Although LPS is intrinsically heterogeneous and over time tends to form aggregates of ~1–4 mDa or greater, smaller LOS or LPS monomers in the range of ~50 to ~100 kDa appear to have little difficulty in transversing ~2.4 nm diameter pores to reach their final destination within the nucleoplasm (Zimmer et al., 1988; Millipore Sigma; Lipopolysaccharides^1^).

### Membrane Disruption by Aβ42 Peptide Oligomers

As recently visualized by atomic force, transmission electron microscopy, mobility-mass spectrometry and liquid surface X-ray scattering there is a remarkable influence of Aβ monomers, short fibrillar Aβ oligomers, globular non-fibrillar Aβ oligomers and full-length Aβ fibrils on lipid bilayer membrane integrity and stability (Bode et al., 2019; Nguyen et al., 2019; Österlund et al., 2019; Vander Zanden et al., 2019). Abundant evidence indicates an Aβ oligomeric fibril-induced reorganization of membrane lipid packing and the induction of membrane destabilization and lipid disorganization by globular non-fibrillar Aβ oligomers (Di Lorenzo et al., 2019; Vander Zanden et al., 2019). Scanning electron microscopy (SEM) and thioflavin-T fluorescence assay have revealed: (i) that LPS and/or LPS-binding protein (LBP) have strong disruptive effects on the structural and biophysical organization of Aβ peptides and amyloidogenesis in Parkinson’s disease (Montagne et al., 2017; Pretorius et al., 2018); and (ii) that LPS strongly induces NF-kB signaling, inflammatory responses, neuroinflammation, the generation of Aβ42 peptides and amyloidogenesis in transgenic murine models of AD (Gu et al., 2018; Jeon et al., 2019; Sheppard et al., 2019). Conversely, as evidenced by atomic force and electron microscopy imaging, short fibrillar Aβ42 oligomers appear to have a profound detergent-like, highly-localized, solubilizing effect on lipid membrane bilayers and this may predispose to hydrophobic interaction with LPS already present in the parenchyma of AD brain (Bode et al., 2019).

### Highly Specialized Features of Neuronal Nuclear Membranes

Used for highly regulated nucleocytoplasmic transport, the nuclear envelope of typical neuronal cells contain about ~10,000 nuclear pore complexes/transporters (significantly more than the ~3,000 nuclear pores of a typical eukaryotic cell), and each ~110 MDa nuclear pore complex (NPC) consists of about ~1,000 nucleoporin proteins (Cooper, 2000; Kabachinski and Schwartz, 2015; Davidson, 2019; Lin and Hoelz, 2019; Sun et al., 2019). The affinity of LPS for any NPC component or any nucleoporin protein is not well understood and is an understudied area of both the neurobiology, microbiology and neuropathology of the human central nervous system (CNS). The perinuclear accumulation of LPS and LPS-mediated envelopment of human neuronal nuclei (Zhao et al., 2017a,b,c), and the restriction of the outflow of neuron-specific information, such as those mRNAs encoding the neuron-specific neurofilament light (NF-L) chain protein and SYN1 (Zhao et al., 2019), underscore the novel pathogenic potential of LPS in supporting dysfunction in neuronal cytoarchitecture and the capacity for efficient inter-neuronal signaling by disrupting SYN1 availability and hence synaptic integrity. In addition, LPS strongly associates with amyloid plaques (Zhan et al., 2018) and perinuclear LPS, and encasement of neuronal nuclei by LPS may also contribute to the biophysical blockage of exit of mRNA through the NPC into the cytoplasm in AD brain (Zhao et al., 2019). Interestingly, using stable isotope labeling of amino acids in cell culture and quantitative proteomics, it has recently been shown that the interactome of the 695 amino acid beta-amyloid precursor protein βAPP695, which is the direct precursor to Aβ42 peptide, interacts strongly with the NPC and nucleoporin proteins in neuronal cells (Andrew et al., 2019). This suggests some novel roles for both βAPP695 and Aβ42 peptide in both NPC function and amyloid peptide processing and generation. The unique phospholipid composition of the inner nuclear neuronal membrane (that encases the genome) and the outer neuronal nuclear membrane that together form the nuclear envelope, their extremely high ratio of phospholipid to cholesterol, the biophysics of nuclear lipid membrane remodeling and lipid raft formation may predispose the neuronal nuclear envelope to the potential interaction between amyloid peptides and LPS, and with NPC nucleoporin proteins.

### Other Interactions Between LPS and Aβ42 Peptides

Lipopolysaccharide (LPS) is a type of prokaryotic glycoconjugate-glycolipid comprised of three major domains: (i) an “O” antigen consisting of an “O polysaccharide”; (ii) a “core” polysaccharide domain (the innermost hydrophilic domain of the three regions of LPS); and (iii) a hydrophobic “lipid A” domain. The “core” polysaccharide domain contains an oligosaccharide covalently attached directly to the “lipid A” moiety and commonly contains sugars such as heptose, 3-deoxy-D-mannooctulosonic acid as well as non-carbohydrate components that include phosphate, amino acids linkages and ethanolamine components characteristic of each Gram-negative bacterial genus and species (Whitfield and Trent, 2014; Tulkens et al., 2018). Both the 50–100 kDa LPS monomer (especially the “lipid A” domain responsible for much of the toxicity of Gram-negative bacteria) and the 4.5 kDa Aβ42 peptide monomer are highly hydrophobic and this alone may favor their mutual interaction with the lipid bilayer of neuronal membranes (National Institutes of Health, PubChem, 2019). As mentioned earlier, Aβ42-based oligomers are highly disruptive toward lipid bilayer membranes, but whether the chaotropic actions of LPS and Aβ42 peptide are additive or synergistic is unknown as their specific interactions are currently not well understood.

## Unanswered Questions

While Aβ42 peptides clearly support LPS entry into neurons it is not clear why neuronal membranes are specifically targeted for these actions and/or why other cellular plasma membranes (lipid bilayers) are not preferred or involved to a lesser extent; perhaps this has something to do with the unique neuronal membrane proteolipid composition and/or the electrical activity of these cell types or other unique neuronal features including Aβ42 peptide-mediated pore formation (see above; Nguyen et al., 2019; Österlund et al., 2019). While LPS has been shown to completely envelope neuronal nuclei in the superior temporal lobe neocortex (Brodmann area A22) in both aging, and especially in AD brain, there is emerging evidence that the end result of this biophysical occlusion of nuclear pores is the restriction of outflow of genetic information, i.e., messenger RNA (mRNA) through these nuclear pores, however some other pathogenic mechanism may be involved (Clement et al., 2016; Lukiw et al., 2018; Zhao et al., 2019; Cornelison et al., 2019). Very recently it has been demonstrated that there is an LPS-induced translocation of cytosolic NF-κB into the cell nucleus (Bagaev et al., 2019) and LPS-induced neuronal hypertrophy (Tellez-Merlo et al., 2019) but these potentially pathogenic and neuroinflammatory outcomes require further investigation. It would be very useful to know at what point an induced disruption along the gut-brain axis and LPS-signaling pathways might be beneficial in the clinical management of AD.

## A Human Brain Microbiome?

There exists the intriguing and enigmatic possibility, as has been suggested for other major organ groups, that the human brain and/or CNS might have its own, as yet poorly characterized microbiome (Bhattacharjee and Lukiw, 2013; Hill et al., 2014a,b; Köhler et al., 2016; Emery et al., 2017; Zhao et al., 2017b,c; Roberts et al., 2018; Zhao and Lukiw, 2018a,b; Zhou and Bian, 2018; Javan et al., 2019; Mazmanian, 2019). Currently, it is understood that microbes can enter the brain and CNS through the BBB which becomes leaky *via* physical damage, disease and/or aging, and/or *via* nerves that innervate both the brain and the gut (Roberts et al., 2018; Javan et al., 2019). Very recent studies indicate the presence of bacteria in the human and mouse brain at the BBB under noninfectious or non-traumatic conditions. Microbes have been identified *via* morphological criteria and ultrastructural imaging analysis with high bacterial counts found in the human hippocampus and prefrontal cortex, but low bacterial counts in other brain anatomical regions such as the striatum (Roberts et al., 2018; Javan et al., 2019). Significantly increased bacterial populations have been observed in association with neurological deterioration in AD brain tissues compared with controls (Emery et al., 2017). Other supportive studies come from investigations involving the human thanatomicrobiome—the microbiome of death—that reflects the post-mortem microbial changes which vary by organ and as a function of time and temperature (Zhao et al., 2017a,b,c; Zhou and Bian, 2018; Javan et al., 2019). Further support for the idea that a microbiome may already be present in the brain arises when considering that microbes (or microbial-derived neurotoxins) in the GI tract would need to travel a significant distance to reach the brain compartments post-mortem vs. the extremely rapid proliferation of bacteria in the brain shortly after death. This might contribute locally to the presence of bacterial-derived neurotoxins such as LPS and other microbial-derived molecules in brain tissues (Hill et al., 2014a,b; Lukiw, 2016a,b; Zhan et al., 2016; Emery et al., 2017). Put another way, post-mortem microscopic examination of the post-mortem brain routinely detects bacteria far more rapidly than the bio-physiological capability of microbes to transit from the GI-tract across the systemic circulation into the brain or other CNS compartments (Roberts et al., 2018; Javan et al., 2019).

## Future Directions

The remarkable affinity of the glycoconjugate LPS, the major component of the outer membrane of Gram-negative bacteria such as *B. fragilis* and *E.coli*, for the neuronal nuclear envelope in human brain cells and tissues was first described just ~2 years ago (Zhao et al., 2017a,b,c; Zhan et al., 2018). The mean abundance of GI-tract-sourced LPS can be increased by either an up-regulation in the biosynthesis of LPS itself or *via* an increase in the number of Gram-negative bacteria capable of generating and releasing LPS in part through the process of dysbiosis. Because both the *de novo* induction of LPS and bacterial division times of ~15–20 min are relatively rapid microbiological-pathological events, it seems also that the production of LPS can be a rapidly undertaken and perhaps even exponential biological event (Raetz and Whitfield, 2002; Whitfield and Trent, 2014; Sweeney and Lowary, 2019). Interestingly, growth rates in the GI-tract microbiome for Gram-negative bacteria have been shown to be dependent on ingested dietary fiber, and the relative proportion of *B. fragilis* in the GI tract can for example decrease 2–3-fold after a fiber-laden meal while a high-fat-cholesterol (HFC) meal has the opposite effect (Heinritz et al., 2016; Huang and Liu, 2019). Indeed, dietary modification by increasing both soluble and/or insoluble fiber intake has been shown to decrease the abundance of *B. fragilis* in the GI-tract microbiome on a time-scale of hours-to-days after the ingestion of the fiber-enriched meal itself (Simpson and Campbell, 2015; Chen et al., 2017; Dhillon et al., 2019; Huang and Liu, 2019; Parada Venegas et al., 2019). Dietary manipulation, probiotics and prebiotic supplementation and increased ingestion of fiber is one research area urgently requiring more study, because the management of diet could yield real and more effective therapies for both the treatment of neurodegeneration and malignancy (Rios-Covian et al., 2017; Poeker et al., 2018; Dhillon et al., 2019; Huang and Liu, 2019). It should be mentioned that under realistic physiological conditions the ~1,800 phyla of bacteria of the GI-tract microbiome are together most likely capable of generating an extremely complex neurotoxic cocktail of exudates, and at this point in time we are analyzing just a very small number of GI-tract derived neurotoxins from a vast neurotoxic pool of huge abundance and bewildering biological complexity (Hicks et al., 2019; Tierney et al., 2019).

## Summary

Over the last few years, the GI-tract microbiome-brain axis has emerged as a focus of increasing interest in the establishment of a neurophysiological and neurobiological basis for age-related, developmental, neurodegenerative, neuroinflammatory and psychiatric disease. The microorganisms which constitute the human GI-tract microbiome have potential to secrete some of the most neurotoxic and inflammation-inducing substances known, including bacterial glycolipid lipopolysaccharide (LPS) from abundant, anaerobic, GI-tract resident Gram-negative bacteria (Whitfield and Trent, 2014; Batista et al., 2019; Patrick et al., 2019; Ticinesi et al., 2019). As a particularly abundant commensal, non-motile, non-spore forming obligatory anaerobic, Gram-negative bacillus of the human GI-tract microbiome, *Bacteroides fragilis (B. fragilis)*, releases an intensely pro-inflammatory species of LPS (BF-LPS), amongst the most pro-inflammatory substances known, that in HNG cells in primary culture induces the pro-inflammatory transcription factor NF-kB (p50/p65) complex (Lukiw, 2016a,b; Zhao and Lukiw, 2018a,b; Batista et al., 2019; Sweeney and Lowary, 2019). LPS translocation into the nucleoplasm and access to neuronal nuclei are greatly facilitated in the presence of Aβ42 peptides (Figures 1, 2). LPS-triggered NF-kB (p50/p65) up-regulation is associated with: (i) the induction of pro-inflammatory, pathogenic microRNA-regulated gene expression programs in the AD brain; these microRNAs have multiple NF-kB (p50/p65) recognition features in their immediate promoters (Pogue and Lukiw, 2018); and (ii) multiple independent laboratories have provided evidence that GI-tract derived glycolipids such as LPS associated with the pro-inflammatory, cytoarchitectural and/or synaptic neuropathology of AD brain and transgenic murine models of AD (Bhattacharjee and Lukiw, 2013; Hill and Lukiw, 2015; Zhan et al., 2016, 2018; Lukiw et al., 2018; Zhao et al., 2019). Many of these noxious biopolymers are potent enterotoxins which can neutralize cadherins and other cell-cell adhesion molecules, inducing leakage through the GI-tract epithelial barrier, which normally is largely impermeable, allowing neurotoxin access to the systemic circulation and subsequent translocation across the BBB (Leshchyns’ka and Sytnyk, 2016; Sweeney et al., 2018; Jeon et al., 2019; Sweeney and Lowary, 2019). Clinically, the detection of these GI-tract microbiome-derived neurotoxins in blood serum may be of prodromal, prognostic and/or diagnostic value as biomarkers for the onset and/or propagation of neurological disease or malignancy; or of forensic value in the determination of temporal aspects of the post-mortem interval (Li and Yu, 2017; Zhou and Bian, 2018).

Lastly, obligate anaerobic bacteria such as *Bacteroides fragilis* make up the largest proportion of Gram-negative microbes in the human GI-tract microbiome. In a recent study of 2,100 human donors, the most recent estimate is that all together microbial constituents of this microbiome harbor at least 22.3 million non-redundant prokaryotic genes in contrast to the 26.6 thousand protein-encoding transcripts of the human genome (Venter et al., 2001; Tierney et al., 2019). Hence, GI-tract microbial genes outnumber host genes by about 840-to-1, which represents staggering genetic complexity (Fields et al., 1994; Venter et al., 2001; Tierney et al., 2019). With this comes a GI-tract microbial proteome of remarkable proportion and speciation that includes highly neurotoxic and pro-inflammatory exudates such as LPS (Hicks et al., 2019; Roy Sarkar and Banerjee, 2019). It is tempting to speculate that we are just scratching the surface of our understanding of the potential impact of these prokaryotic GI-tract microbiome-derived genes and their extruded neurotoxic molecules on our own host gene signaling and expression systems which are likely to have a tremendous impact and relevance to both human health and disease.

## Data Availability Statement

All datasets generated for this study are included in the article.

## Ethics Statement

The animal study was reviewed and approved by Louisiana State University (LSU) Ethics Committee, Louisiana State University, New Orleans, LA, USA.

## Author Contributions

WLi, WLu, TB and YZ performed and analyzed all experiments. WLu wrote the article.

## Conflict of Interest

The authors declare that the research was conducted in the absence of any commercial or financial relationships that could be construed as a potential conflict of interest.

## Footnotes

^1^https://www.sigmaaldrich.com/technical-documents/protocols/biology/lipopoly-saccharides.html (last accessed 5 November 2019).
